# Codon-Optimized Expression and Purification of Truncated ORF2 Protein of Hepatitis E Virus in *Escherichia coli*

**DOI:** 10.5812/jjm.11261

**Published:** 2014-07-01

**Authors:** Fatemeh Farshadpour, Reza Taherkhani, Manoochehr Makvandi, Hamid Rajabi Memari, Ali Reza Samarbafzadeh

**Affiliations:** 1 Health Research Institute, Infectious and Tropical Disease Research Center, Ahvaz Jundishapur University of Medical Sciences, Ahvaz, IR Iran; 2 Department of Medical Virology, School of Medicine, Ahvaz Jundishapur University of Medical Sciences, Ahvaz, IR Iran; 3 Department of Agronomy and Plant Breeding, Faculty of Agriculture, Ahvaz Shahid Chamran University, Ahvaz, IR Iran

**Keywords:** Hepatitis E virus, *Escherichia coli*, Purification

## Abstract

**Background:**

Hepatitis E virus (HEV) is a causative agent of acute hepatitis among people of different age groups and has high mortality rate of up to 30% among pregnant women. Therefore, primary prevention of HEV infection is essential.

**Objectives:**

The aim of this study was to obtain the highly purified truncated open reading frames 2 (ORF2) protein, which might be a future HEV vaccine candidate.

**Materials and Methods:**

The truncated *orf2* gene (*orf2.1*), encoding the 112-660 amino acid of HEV capsid protein sequence, was optimized, synthesized, and cloned into pBluescript II SK(+) vector. After subcloning into expression vector pET-30a (+), a 193-nucleotide fragment was deleted from the construct and the recombinant plasmid pET-30a-ORF2.2 (*orf2.2* encodes 112-608 amino acid sequence of HEV capsid protein) was constructed and used for transformation of *Escherichia coli* BL21 cells. After induction with isopropyl-β-D-thiogalactopyranoside (IPTG) and optimizing the conditions of expression, the target protein was highly expressed and purified by Ni^2+^-chelate affinity chromatography. The expressed and purified protein was analyzed by sodium dodecyl sulfate polyacrylamide gel electrophoresis (SDS-PAGE) and Western blotting.

**Results:**

The subcloning was confirmed by PCR, restriction enzyme digestion, and DNA sequencing of recombinant plasmid pET30a-ORF2.2. The results obtained from optimizing the expression conditions showed that the highest expression of the protein was obtained by adding IPTG at a final concentration of 1 mM at 37℃ for four hours. The expression and purification of truncated ORF2 protein was confirmed by SDS-PAGE and western blotting. SDS-PAGE analysis showed a protein band of about 55 kDa. SDS-PAGE of the purified protein revealed that the highest amount of target protein in elution buffer at the pH of 4.5 was obtained. The yield of the purified protein was about 1 mg/L of culture media.

**Conclusions:**

In this study, the optimized truncated ORF2 protein was expressed in *E. coli* successfully and the highly purified protein was obtained, which can be a potential vaccine candidate and as an antigen in ELISA to diagnose HEV infections.

## 1. Background

Hepatitis E virus (HEV) is the member of the genus *Hepevirus* in *Hepeviridae* family (1). HEV has a single-stranded RNA genome with positive polarity (2). Its genome has three overlapping open reading frames (ORFs), including ORF1, ORF2, and ORF3 (3). HEV has been classified into four genotypes based on the phylogenetic analysis, which are different in terms of geographical distribution and host range (4). Genotypes 1 and 2 are mainly isolated from humans and genotypes 3 and 4 are identified in pigs and other animals (5). Some sources have reported genotype 5 of HEV (1). Most strains belong to genotype 1, which is predominated in Asia and North Africa (6). HEV is a causative agent for acute hepatitis (7, 8). 

HEV infection ranges from moderate to severe hepatitis, but severity of the disease increases with age (8). HEV is endemic in many developing countries, especially in South and Southwest Asia, North Africa, and Middle East (9). Epidemiological data show that one-third of the world's population is infected with HEV (8). HEV is transmitted through fecal-oral route and usually by drinking the contaminated water (7). The mortality rate in general population has been estimated at1% to 15%. HEV infection has a poor prognosis in pregnant women and causes fatal and fulminant infections in pregnant women. The highest mortality rate of up to 30% has been reported in pregnant women and survivors have high rate of miscarriage and premature birth (4). In patients with chronic liver disease, secondary infections (super infection) with HEV can often lead to serious consequences (8). According to what was said, HEV is a serious threat to public health; therefore, rapid diagnosis and primary prevention of HEV infection are required. 

At present, no HEV vaccine has been licensed and producing a vaccine, especially in endemic areas, is a desirable goal (10, 11). Dead and live attenuated vaccines are not available due to the lack of an appropriate culture system for HEV (3, 6). Among the HEV proteins, those which are encoded by *orf1 *gene are nonstructural and therefore, are not available for antibody formation (12). It is not clear that the protein encoded by *orf3 *is structural or nonstructural, but its antibody responses are of short duration. Furthermore, antibodies to ORF3 do not neutralize HEV (13). The *orf2 *gene encodes 72-kDa capsid protein of, which is comprised of 660 amino acids. Viral capsid protein has been studied for future HEV vaccine, as it is an immunogenic component of HEV and is a highly-conserved antigen among HEV species (6). 

Antibodies against capsid protein neutralize HEV *in vitro*, protect primates against HEV, and provide long-term immunity (13, 14), which suggests that it could be used as a possible antigen to diagnose HEV and a promising subunit vaccine against HEV infection in humans. The expressed full-length capsid protein (72 kDa) is not suitable for vaccine production, because the important epitopes are masked and are relatively hydrophobic and insoluble. Short forms (truncated) of capsid protein are very useful for the diagnosis and immunoprophylaxis. The full-length capsid protein (72 kDa) of HEV has several interesting truncated forms, including 53 kDa, 62 kDa, and 56 kDa among which the 56 kDa form is more stable, safe, and immunogenic (15, 16).

## 2. Objectives

The aim of this study was to produce truncated ORF2 protein (55 kDa) of HEV in *Escherichia coli*. Moreover, the major objective of the present study was to obtain highly purified truncated ORF2 protein, which can be a potential vaccine candidate against HEV infection and as an antigen in ELISA to diagnose primary and remote HEV infections in the future.

## 3. Materials and Methods

All kits were purchased from Roche (Germany); T4 DNA ligase and restriction endonucleases were purchased from New England Biolabs (New England Biolabs Inc., USA). Isopropyl-β-D-thiogalactopyranoside (IPTG), polymerase, dNTP, and protein weight markers were purchased from Roche (Germany), Fermentas (Lithuania), or Sigma-Aldrich Corporation (Germany). *E. coli* strain DH5α, *E. coli* strain BL21 (DE3), and the pET30a+expression vector was purchased from Novagen (Novagen Inc., Madison, WI, USA). The nickel-nitrilotriacetic acid (Ni-NTA) Agarose was obtained from Qiagen (Germany). All chemicals were purchased from Merck (Germany) and Sigma-Aldrich Corporation (Germany).

### 3.1. Gene Optimization and Synthesis

The complete nucleotide sequence of the HEV genotype 1 (isolate Sar-55) *orf2 *gene was retrieved from Gen-Bank (accession no. AF444002.1). The genetic codons of wild type truncated *orf2 *gene (*orf2.1*), encoding the 112-660 amino acid of the HEV capsid protein sequence, were optimized using GenScript Rare Codon Analysis Tool software (www.genscript.com). Two restriction enzymes digestion sites including *NdeI* and *XhoI* were placed in the codon-optimized *or f2.1* gene for subcloning into pET30a (+) vector. In order to construct *orf2.2 *(encoding 112-608 amino acid sequence of the HEV capsid protein) from *orf2.1*, the first digestion site for *NheI *was identified at amino acid 608 and the second digestion site was added after stop codons. After the second *NheI* digestion site, 8-His tag and two stop codons were added. For confirmation of our designation, the virtual digestion was done by Clone Manager Basic software version 9 (Sci-Ed Software, Cary, NC, USA) and the digested sequences were aligned with Sar-55 strain using MEGA software version 4.0 (Biodesign Institute, Tempe, AZ, USA). The optimized gene was synthesized and cloned into commercial cloning vector pBluescript II SK (+) by Biomatik Company in Canada (http://www.biomatik.com).

### 3.2. Subcloning and Plasmid Construction

For subcloning the optimized *orf2.1*gene into the expression vector pET-30a (+) (Novagen, Madison, WI, USA), the pBlue script II SK(+) vector carrying optimized *orf2.1* gene (pBluescript II SK-ORF2.1) and the plasmid pET30a+ were both digested by *NdeI *and *XhoI* restriction enzymes (New England BioLab, USA). After *NdeI* and *XhoI* thermal inactivation and analysis in agarose gel, the linearized plasmid and the truncated *orf2* were extracted from agarose gel using agarose gel DNA extraction kit (Roche, Germany) and were used for ligation by T4 DNA ligase (New England BioLab, USA). After ligation, the pET30a-ORF2.1 recombinant plasmid was generated and transformed into *E. coli* DH5α competent cells by electroporation as previously described (17), and selected on Luria-Bertani medium (Himedia, India) agar plates containing kanamycin (50mg/L). 

A number of colonies were assayed by colony polymerase chain reaction (PCR) using plasmid universal primers of T7 promoter and T7 terminator. After choosing the recombinant clones, the plasmid DNA was extracted from the overnight culture by High Pure Plasmid Isolation Kit (Roche, Germany). Then a 193-nucleotidefragment was deleted from the recombinant plasmid pET30a-ORF2.1 by digestion with *NheI*, ligated by T4 DNA ligase, and recombinant plasmid pET30a-ORF2.2 (encoding 112-608 amino acid sequence of HEV capsid protein) was constructed. Then the plasmid DNA was extracted and confirmed by PCR, restriction enzyme digestion, and DNA sequencing. In order to raise protein expression, the recombinant plasmid was transformed into competent *E. coli* BL21 (DE3) cells.

### 3.3. Protein Expression and Purification

A single colony of *E. coli* BL21 (DE3) carrying the recombinant plasmid pET30a-ORF2.2 was cultured in Terrific Broth medium (Himedia, India) supplemented with kanamycin (50 mg/L). The overnight culture was inoculated to fresh Terrific Broth medium with kanamycin (50mg/L) in a 1:100 volumetric ratio and incubated at 37℃ with shaking at 250 rpm until the absorbance at optical density of 600 nm (OD600) reached 0.5. The bacterial culture was induced by adding various concentrations (0.1-1 mM) of IPTG and was grown with shaking during different induction times (2, 4, 6, 8, and 16 hours) and different induction temperatures (37℃, 30℃, and 25℃) to optimize the protein expression. After optimizing the conditions of protein expression, induced cells from 200 mL Terrific Broth were pelleted by centrifugation at 4000 rpm for 20 minutes at 4℃. 

The bacterial pellet was suspended in 50 mL phosphate-buffered saline (PBS), then lysed by three cycles of freeze-thawing in liquid nitrogen and cold water (4℃), sonicated in three ten-second bursts, and centrifuged at 15000 rpm for 30 minutes at 4℃. The pellet was suspended in lysis buffer (6 mol of GuHCl, 20 mM of NaH_2_PO_4_, and 500 mM of NaCl; pH, 8.0) and incubated for 30 minutes at room temperature with shaking to solubilize the inclusion body proteins. The suspension was centrifuged at 15000 rpm for 30 minutes at 4℃. The clear supernatant was collected and used for the puriﬁcation by Ni^2+^-chelate affinity chromatography using His tag in a ratio of 0.5 mL of Ni-NTA agarose slurry (Qiagen) for 100 mL of culture. The agarose-sample suspension was gently agitated at room temperature for 30 minutes to allow protein to bind to agarose and then centrifuged at 1000 rpm for two minutes. The supernatant was removed and the agarose was washed three times with ten volumes of binding buffer (8 mol/L of urea, 20 mM of NaH_2_PO_4_, and 500 mM of NaCl; pH, 8.0). The agarose was then transferred to a column and washed twice with three volumes of wash buffer (binding buffer ata linear gradient pH of 8.0, 6.5, and 6). The protein bound with Ni-NTA agarose was eluted by adding four volumes of elution buffer (binding buffer ata linear gradient pH of 5, 4.5, and 4). The eluate was refolded by dialysis in 500 mL of PBS including 10% glycerol at 4℃ for eight hours. The collected samples were examined by sodium dodecyl sulfate-polyacrylamide gel electrophoresis (SDS-PAGE).

### 3.4. Sodium Dodecyl Sulfate-Polyacrylamide Gel Electrophoresis and Western Blot Analysis

SDS-PAGE was performed according to the procedure described previously (18). For Western blotting, protein extracts separated by SDS-PAGE were transferred to a 0.45 µm pore polyvinylidene difluoride (PVDF) membrane (Roche, Germany) using a semi-dry transfer cell (Bio-Rad, USA). Expression of the truncated ORF2 protein was monitored through immunodetection with anti-His6-peroxidase (Roche) using the manufacturer's protocol. Finally, the target protein was visualized with 3,3'-Diaminobenzidine (DAB) as a substrate (19). Protein concentration was determined by Bradford method (20).

## 4. Results

Codon optimization of truncated *orf2* gene (*orf2.1*) was performed based on *E. coli* favorite codons without changing the amino acid sequence using GenScript software. Analyses of the codon adaptation index (CAI) before and after optimization were performed using this software. Possibility of high protein expression level is correlated with the value of CAI. In other words, a CAI of 1.0 is considered as ideal while a CAI of larger than0.8 is rated as good expression in the desired expression organism. In our study a CAI of 0.85 was determined for the optimized gene. The optimized gene was synthesized and cloned into pBluescript II SK (+) vector. The optimized *orf2.1* gene was subcloned into the expression vector pET-30a (+), generating the recombinant plasmid pET30a-ORF2.1. After subcloning, a 193-nucleotide fragment was deleted from the construct and the recombinant plasmid pET-30a-ORF2.2 was constructed and used for transformation in *E. coli* BL21 cells. 

**Figure 1.**
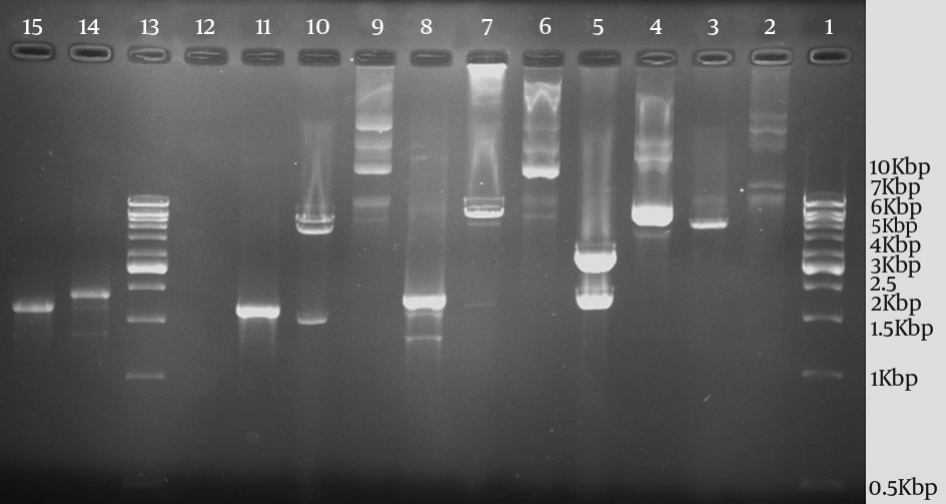
Polymerase Chain Reaction Amplification and Restriction Enzyme Analyses of Different Plasmids by *NdeI* and *XhoI *Restriction Enzymes and Comparison of Undigested and Digested Patterns of Plasmids With orf2.1 and orf2.2 on Agarose Gel Electrophoresis PCR amplification and restriction enzyme analyses of plasmids pBluescript II SK-ORF2.1, pET30a-ORF2.1, pET30a-ORF2.2, and pET30a+ without ORF2.1 by *NdeI* and *XhoI *restriction enzymes. Lane 1, the 1 kb DNA marker; Lane 2, the undigested plasmid pET30a+; Lane 3, the digested plasmid pET30a+; Lane 4, the undigested pBluescript II SK-ORF2.1; Lane 5, the digested pBluescript II SK-ORF2.1; Lane 6, the undigested plasmid pET30a-ORF2.1; Lane 7, the digested plasmid pET30a- ORF2.1; Lane 8, the amplified *orf2.1* gene by PCR (with T7 promoter and T7 terminator primers); Lane 9, the undigested plasmid pET30a-ORF2.2; Lane 10, the digested plasmid pET30a-ORF2.2; Lane 11, the amplified *orf2.2* gene by PCR (with T7 promoter and T7 terminator primers); Lane 13, the 1kb DNA marker; Lane 14, the amplified *orf2.1* gene by PCR; and Lane 15, the amplified *orf2.2* gene by PCR.

The subcloning was confirmed by PCR, restriction enzyme digestion, and DNA sequencing of the recombinant plasmid pET30a-ORF2.2. The size of PCR products on 1.2% agarose gel electrophoresis for *orf 2.1* and *orf 2.2 *genes with plasmid universal primers were 1943 and 1750 bp, respectively, which were consistent with the expected size (Figure 1). The result of sequencing analysis with plasmid universal primers showed that the synthesized gene agreed with what had been designed. A series of expression conditions that differed in induction time, IPTG concentration, and induction temperature were tested to optimize the protein expression. The results obtained from optimizing the expression conditions showed that the highest expression of the protein was obtained by adding IPTG at a final concentration of 1 mM at 37℃ for four hours. After being induced with IPTG, the target protein was highly expressed and purified by Ni^2+^-chelate affinity chromatography. 

**Figure 2.**
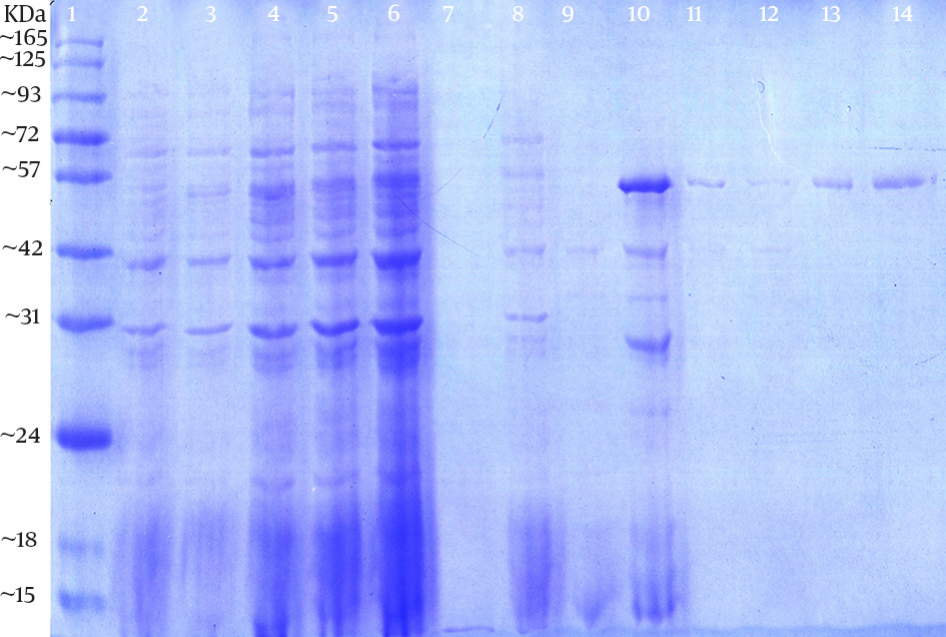
Sodium Dodecyl Sulfate-Polyacrylamide Gel Electrophoresis Analysis of Proteins Expressed bypET30a-ORF2.2 Recombinant Expression Vector in *E. coli* BL21 (DE3) Competent Cells The expressed and purified proteins were analyzed by 10% SDS–PAGE and stained with Coomassie Brilliant Blue R250. The ORF2.2 protein band was seen at about 55 kDa. Lane 1, the prestained protein ladder; Lane 2, the non induced control; Lane 3 to 6, the proteins pattern at two, four, six, and eight hours after induction; Lane 7-9, the washing steps of Ni^2+^ column; Lane 10, purifying Ni^2+^-Denature-5.0 eluate with Ni^2+^ column; Lane 11 and 12, purifying Ni^2+^-Denature-4.5 eluate with Ni^2+^ column; and Lane 13 and 14, purifying Ni^2+^-Denature-4.0 eluate with Ni^2+^ column.

**Figure 3.**
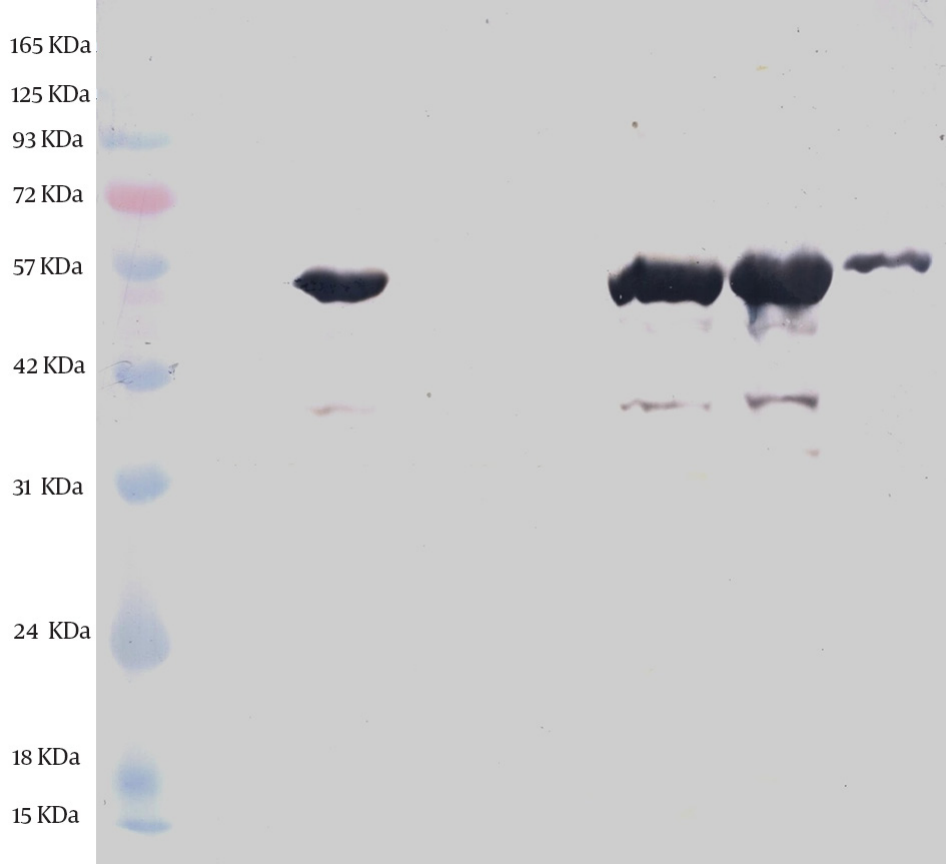
Western Blot Analysis of the Expressed and Purified ORF2.2 Protein in *E. coli* The ORF2 protein band is seen at about 55 kDa. Lane 1, the prestained protein ladder; Lane 2, the noninduced control; Lane 3, Inducing for fourhours; Lane 4, flow-through; Lane:5, wash; Lane: 6 to 8, the first, the second, and the third eluates.

The expression and purification of truncated ORF2 protein was confirmed by SDS-PAGE and western blotting. SDS-PAGE analysis showed a protein band of about 55 kDa, which was in agreement with the expected molecular weight; however, no band was detected in non induced culture. SDS-PAGE of the purified ORF2.2 protein showed that the abundant target protein appeared in the eluate of the elution buffer at pH of 4.5 (Figure 2). The yield of the purified protein was about 1 mg/L of culture media. Western blotting analysis was performed and the presence of the truncated ORF2 protein in *E. coli* BL21 (DE3) was confirmed (Figure 3).

Recently, some studies have indicated that the recombinant proteins are highly expressed by codon optimization (28, 29). Since preferred codons differ in different organisms, there is a direct correlation between host favorite codons and expression level. Rare codons and high-GC contents can decrease or even fail the expression. Therefore, the expression level can be improved by codon optimization and lowering the GC content (30). There are some reports on the traditional way of expression of the HEV structural gene (19, 22); however, there is no report on the optimized expression of this protein. In the present study, the result of expression indicated that the optimized gene was expressed in *E. coli* effectively and the well expression level of the protein was obtained through gene optimization.

The plasmid pET30a+ was used in this study for subcloning. The pET system is one of the best systems for the cloning and expression of recombinant proteins in the *E. coli* BL21 host cell (31). In this study, the results showed that the highest expression of the optimized truncated ORF2 protein was induced by adding IPTG at a final concentration of 1 mM for four hours with shaking at 37℃. The target protein was obtained through purification by Ni^2+^-chelate affinity chromatography using Histags. Some studies have demonstrated the use of metal-aﬃnity chromatography for the puriﬁcation of recombinant proteins. In their studies, highly purified recombinant proteins were obtained through purification by metal-aﬃnity chromatography (23, 27, 32). 

Metal-aﬃnity chromatography takes only a few hours and gives the biologically active protein of high purity (32) since Histag has no effect on the structure, folding, and function of the protein and facilitates selective binding of the expressed protein to a Ni^2+^-affinity column (33). This procedure is also cost-effective since the Ni–NTA agarose is regenerable and can be used several times (32). Finally, the expressed and purified proteins were analyzed by 10% SDS-PAGE.SDS-PAGE analysis showed a protein band of about 55 kDa; it also showed that the abundant target protein appeared in the eluate of elution buffer at pH of 4.5. The yield of the purified protein was about 1 mg/L of culture media. Western blotting analysis was performed and the presence of the truncated ORF2 protein in *E. coli* BL21 (DE3) was confirmed. 

In conclusion, considerable amount of the highly purified protein was obtained, which can be a potential vaccine candidate against HEV infection and as an antigen in ELISA to diagnose primary and remote HEV infections in the future; however, the evaluation of the immune responses is necessary in further studies.
